# Promoting positive youth development in rural communities: Integrating social work, psychology, and education

**DOI:** 10.1371/journal.pone.0309989

**Published:** 2024-09-20

**Authors:** Jiawei Ren

**Affiliations:** Student Affairs Department, Zhejiang Gongshang University Hangzhou College of Commerce, Hangzhou, China; Nanyang Technological University, SINGAPORE

## Abstract

Considering the peculiar socio-cultural background and developmental obstacles encountered by rural youth in China, the study examines the necessity of adopting an integrated strategy that brings together social work, psychology, and education to promote positive youth development. This research intends to fill the gap by explaining the impact of these factors on community engagement and youth development in China. Targeted programs were also suggested according to the needs of rural youth in China. The respondents of the study comprised 350 young people, whose age ranged from 15 to 24 years, living in different rural areas of the country. The structured questionnaire was designed to collect the data using a convenience sampling technique. Structural Equation Modeling (SEM) was applied as the analysis tool using IBM SPSS AMOS software. The results show that social work and education have a significant impact on community engagement and positive youth development. The findings also reveal that psychology positively influences community engagement. Community engagement was seen to mediate the relationships between social work, psychology, education, and positive youth development. The policymakers and practitioners can fully use the interrelationships between social work, psychology, and education to create a more comprehensive approach that considers the specific characteristics of rural youth in China. Additionally, highlighting community engagement as a mediator also explores the opportunity for bottom-up initiatives and community efforts to instigate favorable youth outcomes in the countryside.

## 1. Introduction

Youth development, which has profound implication for the fortunes of both local communities and the country as a whole cannot be undermined in the Chinese rural areas [1]. Therefore, multidimensional way of youth development is essential. Lerner et al. [2] stated that cities are usually endowed with immense resources and facilities. However, rural areas face problems such as inaccessibility to education, health, and jobs. As such, the youth development programs in these areas should use the creative approaches and integrate some disciplines particularly social work, psychology as well as education and community engagement as mediator. Being the majority of the population in China, youth encounter some unique challenges. The education in the rural areas is often challenged by resource constraints which is reflected by the under achievement and the low vocational skills [3]. In addition, the shortage of mental health services and psychological support is also a matter that is faced by rural youth, and thus results depression, anxiety and drug abuse [4]. Moreover, the social and economic gaps and the increasing rural-urban migration level destroy the sense of place and self-worth of the youth in the rural areas [5].

The combination of social work, psychology and education in rural areas provide vibrant services that help the complex youth needs. Social work strategies to be effective by linking the youth with communities resources, protecting their rights, and making them strong [6]. The psychological interventions such as counseling and psychoeducation can empower the rural youth to face the challenges using proper coping strategies [7]. Finally, the educational initiatives which are tailored to the specific needs of rural communities ensures young people to be knowledgeable and capable of personal and social economic growth [8]. The success of these rural-centered initiatives is driven by the commitment of the rural community. Community engagement, as a crucial catalyst, overcomes the disconnection between interventions and their application within the diverse rural conditions [9]. Through support from community members such as parents, teachers, local leaders and youth themselves, different intervention tools can be formulated to meet community’s cultural values, traditions and aspirations. Lastly, active community engagement provides a feeling of belongingness and a sense of shared responsibility which in turn motivates rural residents to become the change makers in their communities [10]).

However, successful community engagement entails a complex comprehension of the social interactions and power structures that become entangled within rural areas. Traditional hierarchical structures and cultural conventions might shape the way resources are distributed as well as the decision-making, doing good approach that promotes equity, transparency, and collaboration [11]. Also ensuring the issues of trust, communication, and participation are being exhausted to secure the meaningful engagement and continued impact is imperative. Prior research tends to concentrate on the urban areas, ignoring the issues confronting and opportunities available for rural youths in China. To address these gaps, this research will explore ways in which integrated approaches involving social work, psychology, and education improve the development of youth in rural areas. Furthermore, the research reveals the importance of culturally appropriate interventions that consider regional variations and other key demographic factors affecting youth development in various rural Chinese communities. Finally, this study intends to guide policy and practice interventions intended to boost the welfare and resilience of rural youth in China, hence contributing to the social sustainable development of rural communities as well as the country in general.

## 2. Literature review

Positive youth development in rural communities has become prominent among the researchers and practitioners, which has generated a vast literature on various methods and interventions.

The process by which young people acquire social, emotional, and cognitive abilities is known as youth development. Family, peers, schools, and communities all have an impact on this development, which aims to produce capable, accountable, and contributing members of society who can effectively traverse life’s problems [2]. Positive Youth Development (PYD) focuses on skill development, positive connections, active involvement, and fostering young people’s inherent abilities. PYD seeks to empower young people, promote their general well-being, and allow them to actively engage in community life and make constructive contributions to their communities [12].

According to Wu et al. [13], the contribution of social work in the rural areas to the solving of systemic inequalities, the building of social systems, and the promotion of individual and community empowerment also cannot be overlooked. Aware of the peculiar challenges faced by the small communities, these measures mostly adopt a blended approach that involves fortifying resilience and promoting overall wellness among the rural youth. The community-based strategies are at the core of these interventions; they draw from the available resources and networks within the settings. Social workers, in particular, identify and use strengths that exist in the community for the development of long-term solutions addressing conditions that are peculiar to rural youth [14]. Rural area social workers are the advocates for marginalized communities. They stand for the voiceless and fight for policies and programs that will meet the needs of the rural community [15]. Through the demonstration of the distinctive issues faced by rural areas and ensuring that the services and resources are fairly distributed, social workers emerge as a critical agent of social justice and community development. The development of social work in China has been growing trend which reflects the social enlightenment of Chinese culture. Historically, the Chinese social work has its origins in Confucianism with the bases of community mandate which provide for collective welfare and cooperation [16]. More recently, the Chinese government has begun to appreciate the value of professional social work in handling social problems. The policies like the community construction have played a significant role in the development of appropriate social work practices for the community. The community work in China stresses on organizing local community resources to obtain cooperation in addressing local issues. The Chinese model of community work pays more attentions to the mobilization of the collective power and the coordination of efforts for achieving common goals [17].

Rural youth psychological wellbeing is now a major problem with most researchers showing a high prevalence of mental health disorder even compared to the urban counterparts [18]. Rural youth now suffer particularly from depression, anxiety and substance abuse with the social factors in rural environments being the significant root of the problem. Social isolation is among the most important factors which result in an increased vulnerability to mental health issues in rural communities. Geographic isolation and low concentrations of population make social lives limited in rural area, increasing lonely and isolated feelings among rural youth [19]. The scarcity of the economic opportunities in the rural areas further worsen the already existing stress factors and leads to the feelings of being hopeless and despair. Also, the insufficient provision of mental health services in rural communities promotes the aggravated problems that rural youths face. Lack of sufficient mental health professionals and resources, along with the stigmatization of mental health challenges, poses major hurdles in getting people to seek help and get appropriate treatment [20]. Such problems demand precise, tailor-made psychological interventions that are consistent with the expectations of the youth in the rural areas so that they are efficient in mental health promotion.

Education aims not only for personal success but also for general prosperity and for the fortitude of the youths from rural regions as well [21]. Education is seen to be so important but the access to and quality of education are not the same in the rural areas. Studies have highlighted the need for educational programs that are in tune and responsive to the challenges and situations that exist in the rural areas. These problems are becoming increasingly recognized in the field of education as more educators are adopting different approaches to the creation of educational programs that cater for the diverse needs of rural communities [22]. These programs are made to be the main tools that shape society where the capabilities and potential of the rural youth are developed and harnessed.

Jackson et al. [23] states that community engagement is a key factor that promotes positive youth development in small towns. Research has only proved the profound effects of social participation methods that place the community members in the lead position to make decisions and to design programs that truly respond to the needs of the youth in the rural areas. The principle of building strong relationships with a community lies in the principle of inclusivity which ensures that different voices in the community are included in the decision-making process. The sense of ownership and accomplishment will be built in the community members, including the adolescents, who took part in the implementation, organizing and evaluation processes [24].

**Fig 1:**
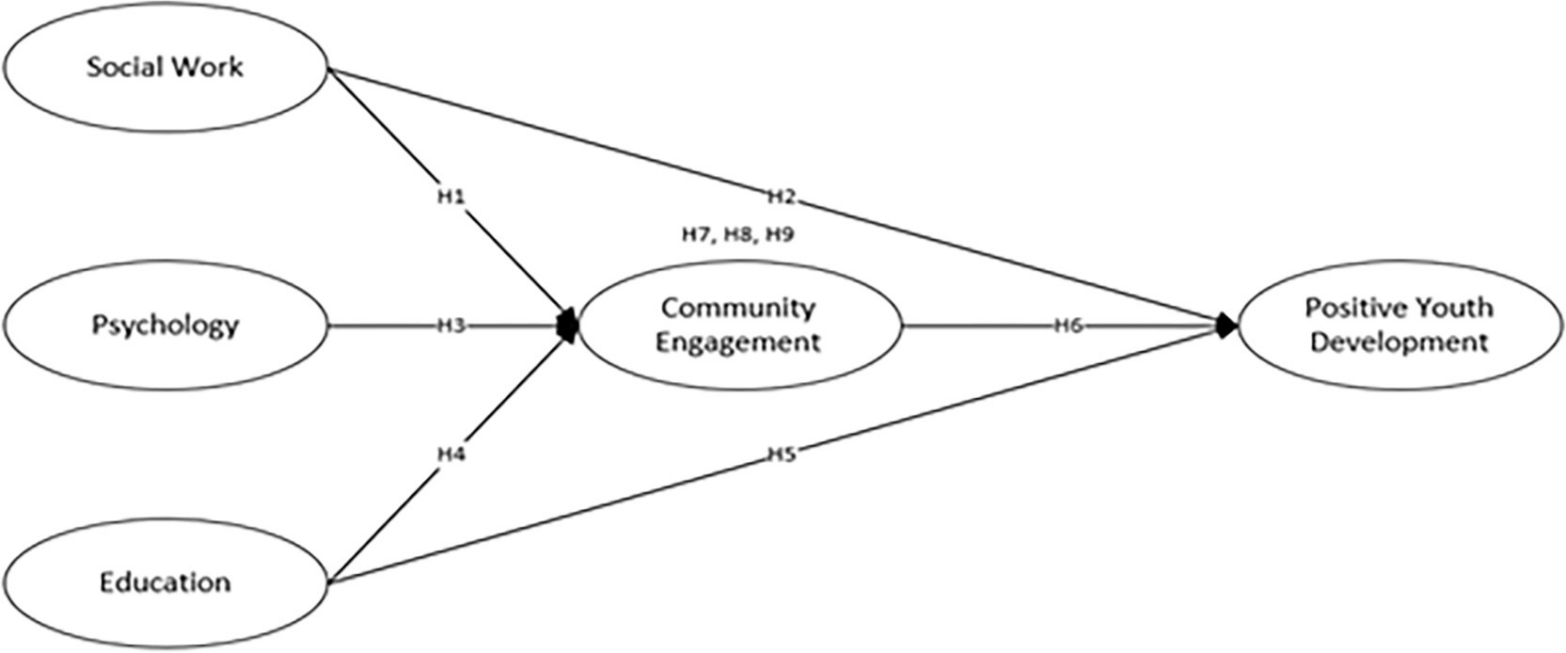
Research framework.

Therefore, this study integrates social work, psychology, education, and community involvement for the purpose of enhancing youth development in rural China. Through targeting the underlying challenges as well as using the inherent strengths in these communities, interventions can empower the youth rural community to succeed and fully take part in their communities and society at large. The research framework is displayed in Fig 1.

### 2.1. Research hypothesis

#### 2.1.1. Social work

McFadden et al. [25] states that social work focuses on improving the well-being and quality of life in both individual and group environments. It covers a wide variety of activities, designed to address social problems, to support social justice, and to strengthen the position of the deprived or weak groups of society. This study, therefore, involves social work by applying professional knowledge, skills, and values to provide the relevant services to the youth living in the rural communities. Social workers can do the practical services for youths like counseling, case management and advocacy which aim at the well-being and development of youth [6]. They may also collaborate with families, schools and community agencies to develop positive environments with easy access to resources that lead to positive youth development. Community engagement is all about bringing together and working with individuals, groups, organizations, and stakeholders in a community to deal with issues, make decisions, and advance positive changes [26]. It is based on the principles of full participation, dialogue, and joint governance to solve common problems and improve the well-being of the community. In terms of this, community engagement is about the role of different people i.e. youth, family, educators, social workers, psychologists, and community leaders’ joint effort with the purpose of positive youth development [2]. Such contribution can be engaging in community-based programs, partnerships between different sectors and activities oriented to address the specific needs of the youth in rural areas.

Social work interventions become drivers of community engagement efforts geared towards the improvement of the well-being of the rural youth. By evaluating community needs and bringing together stakeholders to develop strategies, social workers ensure community involvement within youth development programs. Social workers are middlemen between young people, their families, schools and community institutions, and by establishing collaborative linkages they develop holistic approaches to solve the complex issues of youth [27]. Through social work principles of inclusivity, empowerment, and collaboration communities unite to collaborate in identifying their strengths, resources, and solutions which help to support the wellbeing of the youth in the community. Social work interventions serve as a strategy for capacity-building of communities through leadership development, building resilience, and stimulating ownership and responsibility for the development of youth among the community members. Involving social workers in community engagement activities makes a way for the establishment of sustainable networks and infrastructure which continue to support positive youth outcome even when the specific programs or interventions are over [28].

H1: Social work significantly influences community engagement

Positive youth development is an approach that concentrates on enhancing the strengths, the capacity, and the power of youths to do well and live an excellent life [29]. It also focuses on promoting development of young people emotionally, socially, cognitively, and physically. Tailored social work interventions targeting the specific needs of the rural youth go beyond in promoting their better development by directly providing needed support, resources, and guidance [30]. Rural youth are enabled to develop resilience and coping strategies for life’s challenges through one-on-one and group counseling, thus their positive life outcomes will be enhanced. Social workers are important in linking the rural youth with community resources as well as mentorship programs and extracurricular activities through which the youth can engage in skill-building, leadership development, and growth of their personalities [31]. By eliminating systemic barriers and advocating for policy changes which promotes equity and access to education, healthcare and social services, social workers make the rural youth more robust to excel and reach their full potential. Social work, which embraces a holistic approach considering the many contributing factors of family, school, peers, and community, allows the practitioners to deal with complex issues and consequently, implement holistic interventions that encourage positive results for the youth in rural areas [32].

H2: Social work significantly influences positive youth development

#### 2.1.2. Psychology

Psychology is the scientific field of the study of mind and behavior. It covers a large number of issues such as cognition, emotion, motivation, personality, development, social relations, mental health etc. [33]. Psychologists are utilizing different types of research methods and techniques to understand how individuals perceive, think, feel, and behave not only individually but also in social contexts. Psychology plays a pivotal part in revealing the psychological processes and developmental requirements of the youth in rural areas [34]. The knowledge of the psychological dynamics of the community, the social norms and the group behaviors contribute to the success of community involvement in advocacy of positive youth development in rural areas. Psychologists act as a skilled workforce conducting needs assessments, determining barriers to community engagement as well as designing interventions that target cognitive, emotional, and motivational factors causing involvement deficits in members of the community especially youth [35]. Through implementation of research and program evaluation, psychologists share the important information about the efficiency of different community engagement ways, thus, modifying strategies, allocating resources, and enlarging successful developments. Psychological principles of persuasion, communication, and conflict resolution play an important role in making community engagement efforts more effective via building trust, consensus and dialog among the stakeholders with different perspectives and interests [36]. The psychologists’ approach through promoting an inclusive, empowering, and collectively engaging culture within communities would be an encouragement of active involvement, collaboration, and joint efforts in the common goals of youth development and community prosperity [37].

H3: Psychology significantly influences community engagement

#### 2.1.3. Education

According to Ismail et al. [38], educating is a systematic way of promoting knowledge, skills, values, beliefs, and habits through the various means like teaching, training, storytelling, research, and experience. It is a defining feature of human trajectory and it plays a key role in molding persons and societies. Educational actions include all sides of life of rural youth. It advocates for culture-based pedagogy, location-based interventions, and multi-sectional participation for the youth to be empowered and involved in a rural society [39]. Education of youth in rural areas give them the ability to overcome the challenges they encounter and contribute to community development in the long run. When education is participatory and design-infused with local contexts, it becomes a powerful tool in developing active citizenship and community involvement as the outcomes have already proved in the rural areas [40]. Education is the catalyst of integration and development and this is the foundation of collective prosperity and long-term progress. The effect of education on the community engagement in rural areas is crucial as it can help empower the youth, create local leaders and promote a culture of active participation hence revitalizing and strengthening the local communities [41].

H4: Education significantly influences community engagement

Education is an important factor that influences positive youth development through knowledge acquisition, skills development, and personal growth [2]. Education therefore equips the youth with the intelligence skills, problem-solving ability, and endurance which are the building blocks of a positive youth development. The quality education gives youth the chance to discover their passion and chase their dreams which is very critical for their development and general well-being [42]. Besides all the academic knowledge, youth acquire social and emotional intelligence which allows them to build healthy relations, self-awareness and empathy that are relevant for better development. Education investments not only enhance cognitive development but also have the ability to increase socio-economic mobility, thus driving youth out of poverty cycle and making them succeed in life [43]. The educational environment is probably the most critical factor in the development of youth belonging, identity formation, and personal agency that are key elements of positive youth development. Educational experiences, formal and informal, which influence the youth attitudes, values and beliefs, are used in the decision-making processes, which, in turn lead to a good development trajectory [44]. The successful collaboration among teachers, families, and communities builds up the supportive learning environment in which youths discover and develop their qualities, interests, and dreams, which leads to holistic positive development. Education serves as a catalyst for personal empowerment, building resilience, chasing goals, and giving back to community and society as a whole. Identifying the multifaceted function of education in the formation of young lives calls for comprehensive strategies that are integrative in nature in order to ensure positive youth development in the rural areas [45].

H5: Education significantly influences positive youth development

#### 2.1.4. Community engagement

Community engagement plays a vital role in building positive youth development through its supportive platform that helps in creating a sense of belonging and offering chances for meaningful involvement. The participation of young people in community activities, like volunteering, mentoring and civic engagement, positively correlates with youth development outcomes, e.g., better academic performance, improved social skills, and higher self-esteem [46]. In rural areas, which often involve resource scarcity, the role of community engagement is more critical in dealing with isolation and economic discrepancies, as it gives youth an opportunity to be empowered both personally and collectively. Youth are not just beneficiaries of the community engagement initiatives but also proactive young people who are actively involved in the development of their communities and at the same time are developing essential life skills, for instance, leadership, teamwork and problem solving [47]. The integration of social work, psychology, and education in the promotion of community engagement activities are the foundation of a synergistic approach as it multiplies the impact in positive youth development through the addressing of the multifaceted issues and the promotion of holistic development. The youth who willingly involve themselves in community activities are more likely to emerge as resilient, responsible, and with a sense of purpose even into their adulthood. Community engagement initiatives serve as platforms for youth to explore their interests, develop skills and discover their passions in a safe environment [48]. The process boosts the confidence of the youth and prepares them for the challenges ahead as they strive to achieve their goals. Youth should be given a chance to be involved as a decision-makers in the community decision-making processes so that their voices are heard and also cultivates a sense of ownership and responsibility that makes a culture of inclusion and social justice in the rural communities [49].

H6: Community engagement significantly influences positive youth development

#### 2.1.5. Community engagement as a mediator

Community engagement represents a crucial link between social work and positive youth development within rural environments. Social work interventions attain more traction in community-based programs through active involvement and community engagement, in turn facilitating a condition where youth progress organically and subsequently this stresses the essentiality of community engagement as a mediator [50]. The inclusion of community engagement tactics into social work practices shows the practitioners’ capability to cultivate a sense of ownership and participation of the local stakeholders that will lead to collaborative activities, which eventually results into rural area youth development. Community engagement allows for social work professionals to explore the community’s special traits and resources. This contributes to the success of the interventions and, in the long run leads to development of positive youth trajectories [51]. In the symbiotic relationship between social work and positive youth development, engagement of the community becomes a critical mediator that enables the exchange of knowledge, resource, and support systems needed for holistic growth and resilience development in rural youth.

H7: Community engagement mediates the relationship between social work and positive youth development

Community engagement can help translate psychological principles into concrete approaches that work in rural contexts and are responsive to the unique needs and cultural practices of these communities. Such approaches nurture positive youth development and the sense of belonging and social capital of young people in this environment. The community participation is a bridge that facilitates application of psychological theories in real life situations by making the theories culturally appropriate and contextual in such a way that the interventions are meaningfully impactful and lead to positive youth outcomes [52]. Community engagement serves as a medium that bridges the experience of psychologists into community-oriented programs that are designed specifically to meet the needs and utilize the potentials of rural youth, thereby creating resilience, self-efficacy, and psychological well-being. Embracing community resources and local intelligence allows engagement campaigns to unlock the distinctive assets of rural settings that nurture protective factors integral for positive youth development, eventually building a resilient future for communities to thrive [53].

H8: Community engagement mediates the relationship between psychology and positive youth development

The inclusion of community stakeholders in education-related activities like mentorship programs, extra-curricular activities, and community services, creates a supportive environment for the rural youth development. The community members’ engagement in educational practices is one of the factors that bring up the feeling of belonging and collective responsibility, providing youth with a wide range of role models, resources, and networks which are valuable to the development of their social, psychological, and mental abilities [54]. Working together with educational institutions and communities can help in overcoming challenges for developing positive youth that include poor access to resources and or opportunities. Therefore, more equitable paths will be created for rural youth to grow academically, socially, and emotionally. Community engagement serves as the catalyst that ignites the youth to become responsible leaders in their communities [55]. It develops leadership skills, a feeling of civic duty and ownership over their development trajectories, which is central to the youth’s overall success and wellbeing.

H9: Community engagement mediates the relationship between education and positive youth development

## 3. Methodology

### 3.1. Research design

The study uses the quantitative method to assess the role played by the integration of social work, psychology, and education in improving the situation of rural youth development in China. A cross-sectional design was used to collect data from a large and varied group of the youth living in rural areas. The study sampled people from different geographical areas and backgrounds to increase representation among the groups.

### 3.2. Questionnaire development

The structured questionnaire used in this study was developed based on a comprehensive literature review of youth development, social work, psychology, education, and community engagement. The questionnaire development went through several stages. First, an extensive literature search was carried out to identify core factors and the scales used in previous studies pertaining to youth development in rural settings. Drawing on the literature review, an item pool was made of potential items in order to contain the constructs of interest. They try to answer the issues faced by rural youth in China and how they grow into the specific society. The chosen pool of items was checked by the expert review panel consisting of the scholars and the practitioners in social work, psychology, and education fields. The feedback was used to improve the questions that they were easy to understand, meaningful and appropriate. A pilot study was carried out on the small number of adolescents from the rural area to assess the clarity, simplicity and the suitability of the questionnaire items. In the light of the feedback the questionnaire was revised to improve its credibility and reliability. Following expert reviews and pilot testing, the final version of the questionnaire was made and for the main study purposes.

### 3.3. Sampling strategy

The process of collecting data took a multi-staged approach to ensure fair and proper representation of the rural youth in China This approach involved two distinct stages: purposive sampling and then convenience sampling. The purposive sampling was applied for the initial recruitment of participants from a variety of rural communities in different Chinese regions. The researchers established the purposive sampling technique which enabled them to focus on specific population groups that fell within defined congruent elements such as age range, geographical location and socioeconomic status. This step was therefore aimed at portraying the variety of opinion and life experience of rural youth. Convenience sampling was used as the next step in gathering data. In this stage, the recruitment of more volunteers from the list of shortlisted rural communities was carried out depending on their accessibility and availability. The convenience sampling proved to be the solution to the problem of a large number of people not available for the study purpose. Therefore, a convenience sampling technique was applied in order to supplement the initial purposive sampling approach.

### 3.4. Data collection

The trained research assistants reached out to potential participants in the selected rural communities and furnished them with information on the study objectives, procedures, and voluntary nature of participation. Informed consent from each participant was obtained before they filled the questionnaire. The participants were guaranteed of the confidentiality and anonymity of their answers. They were given the choice to complete the questionnaire either in paper form or electronically, to suit their preference and based on their access to technology.

### 3.5. Participants

The total study participants were 350 young people aged between 15 and 24 from different rural areas of China. If we talk about age of the respondents, 20% of the respondents were 15–17 years old and 30% was 18–20 years. The remaining 50% belonged to the age group of 21–24 years. Gender of the respondents is explained as 60% males and 40% females. In response to educational background, 30% of the respondents were school students, and 15% of them finished primary education; the remaining 40% of them completed secondary education. 10% among them were college/university students while 5% were not attending any educational institutions for the time being. The respondents were fairly distributed across the different regions in China with the north having 20% of the total, the south having 30%, the east having 25% and the west having 25% as well. About 50% of the respondents came from families with a low socio-economic class while 25% belonging to the middle socio-economic class. Families with a high socio-economic status represented 25% of the respondents. In response to socio-economic status of the respondents, 35% were engaged in agricultural labor, 25% were part of non-agricultural labor, 30% were students, while only 10% were unemployed. According to the family structure of the respondents, 40% were from nuclear families, 35% of them belonged to extended families, 15% were from single-parent household, and 8% were either orphans or under the care of relatives. Only 2% said they lived with other family structures.

### 3.6. Data analysis

The structural equation modeling (SEM) techniques were used to analyze the collected data with the software IBM SPSS AMOS. Through SEM, it is possible to investigate the deep connections among interacting factors and test theoretical models [56]. The study examined the effects of community engagement and positive youth development through the interconnected roles of social work, psychology, and education, with community engagement being the possible mediator factor.

## 4. Findings

The measurement model was employed to evaluate the study items’ reliability and validity. The hypotheses were tested by the structural model.

### 4.1. Measurement model

**Fig 2:**
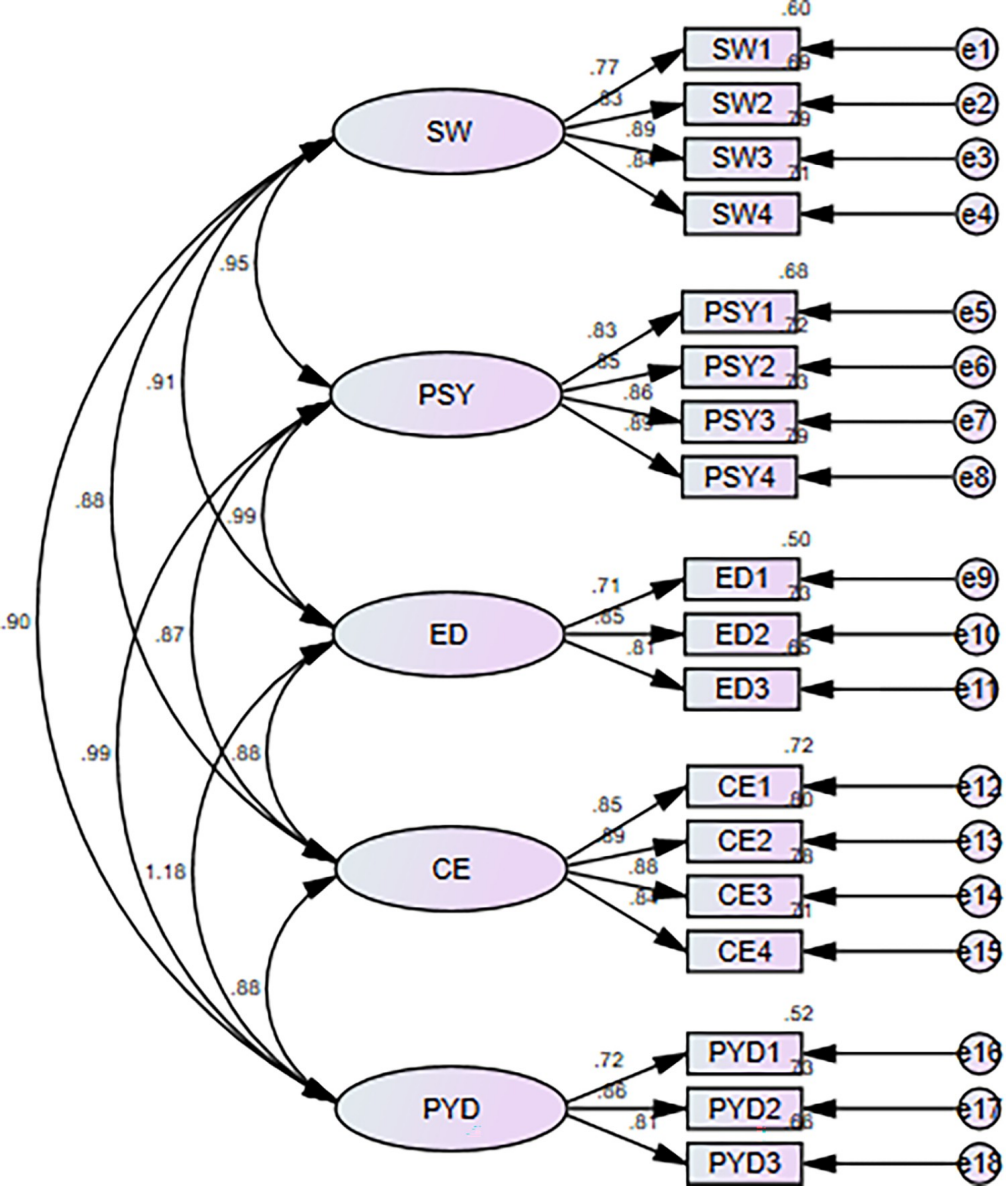
Measurement model.

The confirmatory factor analysis (CFA) was applied in AMOS to assess the measurement model. Table 2 shows that all the factor loadings are higher than the cut-off value of 0.6 [57]. CMIN/DF, CFI, NFI, GFI, RMR, SRMR and RMSEA were computed to assess the model fitness. The values for these indices were found to be within the accepted range [58, 59]. All CFA fit indices are demonstrated in Table 1. This measurement model is illustrated in the Fig 2.

**Table 1:** CFA Fit Indices.

Measure	Abbr.	Recommended Values	Scores
Chi-square/df (CMIN/DF)	X^2^/df	< 3.0 *	1.883
Comparative Fit Index	CFI	> 0.90 *	0.962
The Normed Fit Index	NFI	> 0.90 *	0.917
Goodness of fit	GFI	> 0.90 *	0.909
Root Mean Square Residual	RMR	< 0.08 *	0.053
Standardized Root Mean Square Residual	SRMR	< 0.08 *	0.039
Root Mean-Square Error of Approximation	RMSEA	< 0.08 *	0.057

X^2^/df = Chi-square/df (CMIN/DF), CFI = Comparative Fit Index, NFI = The Normed Fit Index, GFI = Goodness of fit, RMR = Root Mean Square Residual, SRMR = Standardized Root Mean Square Residual, RMSEA = Root Mean-Square Error of Approximation

#### 4.1.1. Reliability

The reliability of constructs was evaluated using the Cronbach’s alpha (α) and composite reliability (CR). Table 2 shows that the Cronbach’s alpha of all the research constructs is higher than the recommended 0.70 threshold illustrated by Nunnally & Bernstein [60]. Composite reliability (CR) values that ranges from 0.834 to 0.924 are higher than the standard value of 0.70 [61]. That’s why they are viewed as reliable and appropriate indicators for the intended constructs.

**Table 2:** Construct reliability and validity.

Constructs	Items	Loadings	α	AVE (> 0.5)	CR (> 0.7)
Social Work	**SW1:** Social workers help me deal with my private problems and concerns	0.772	0.831	0.696	0.902
	**SW2:** Social work support has changed my approach towards life as I have learned to utilize important life skills such as problem-solving and decision making	0.831			
	**SW3:** I am grateful for the social workers who are in my community and actively advocate for our rights and well-being as young people	0.887			
	**SW4:** In my opinion, social work services are paramount to developing harmonious environments and social ties in my community	0.844			
Psychology	**PSY1:** I hold a high sense of self-esteem and confidence in my skills	0.828	0.884	0.733	0.917
	**PSY2:** Psychological counseling has taught me how to comprehend and to deal with my emotions	0.850			
	**PSY3:** I am always on a lookout for ways in which I can grow as an individual and get better as a person	0.857			
	**PSY4:** I think mental health is a priority and that we should get the right help when required	0.889			
Education	**ED1:** Participating in quality education is what keeps the youth socially and economically strong	0.707	0.814	0.626	0.834
	**ED2:** I find myself excited to strive for academic success and reach my educational aims	0.854			
	**ED3:** Educational opportunities in my hometown are a major source of networking and inspiration for me	0.807			
Community Engagement	**CE1:** I take part in community events and projects actively and therefore I help my community stay healthy	0.846	0.895	0.751	0.924
	**CE2:** I develop a strong sense of community and relatedness	0.895			
	**CE3:** Community organizations and programs give youth meaningful access to help and guidance	0.882			
	**CE4:** I am a person who values volunteering and contributing to my community. My aim is to bring about positive social change	0.844			
Positive Youth Development	**PYD1:** I look at the future with optimism and believe in myself and my ability to accomplish my objectives	0.721	0.827	0.635	0.839
	**PYD2:** I gained the strength to make the right choices and take charge of my life	0.856			
	**PYD3:** I believe I am an individual with distinct identity and goals	0.809			

#### 4.1.2. Convergent and discriminant validity

AVE (average variance extracted) criterion was employed to measure the convergent validity of the scale items. Table 2 displays the AVE values of the constructs, and all exceed the 0.50 criterion [62]. Therefore, these findings confirm convergent validity. The Fornell-Larcker Criterion (FLC) [62] and the Heterotrait-Monotrait Ratio (HTMT) (Henseler et al. [63] were used to test the discriminant validity. The outcome of FLC provided evidence that the model was designed for all constructs. The discussion started by Fornell and Larcker criterion critics eventually paved the way for the rise of the reliance on the HTMT. The results demonstrated that the all the HTMT values were under the acceptable range of 0.90 [64]. Table 3 presents a HTMT matrix.

**Table 3:** HTMT.

	Social Work	Psychology	Education	Community Engagement	Positive Youth Development
Social Work					
Psychology	0.35				
Education	0.21	0.23			
Community Engagement	0.47	0.22	0.38		
Positive Youth Development	0.44	0.41	0.36	0.25	

### 4.2. Structural model

The study hypotheses were examined with AMOS structural model. Hair et al. [61] argues that CMIN/DF, CFI, NFI, GFI, RMR, SRMR and RMSEA should stay in the satisfactory level by means of its structural model. The numerical for the parameter metrics was within range of those recommended as shown in Table 4. The R^2^ value was 0.51 for community engagement and 0.63 for positive youth development.

**Table 4:** Structural model fit indices.

Measure	Abbr.	Recommended Values	Scores
Chi-square/df (CMIN/DF)	X^2^/df	< 3.0 *	1.875
Comparative Fit Index	CFI	> 0.90 *	0.959
The Normed Fit Index	NFI	> 0.90 *	0.904
Goodness of fit	GFI	> 0.90 *	0.907
Root Mean Square Residual	RMR	< 0.08 *	0.052
Standardized Root Mean Square Residual	SRMR	< 0.08 *	0.038
Root Mean-Square Error of Approximation	RMSEA	< 0.08 *	0.054

X^2^/df = Chi-square/df (CMIN/DF), CFI = Comparative Fit Index, NFI = The Normed Fit Index, GFI = Goodness of fit, RMR = Root Mean Square Residual, SRMR = Standardized Root Mean Square Residual, RMSEA = Root Mean-Square Error of Approximation

The structural model was used for the hypotheses testing. Table 5 shows that there is positive association between social work and community engagement (β = 0.563, t = 13.731, p < 0.001), as well as between social work and positive youth development (β = 0.631, t = 5.214, p < 0.001), therefore, H1 and H2 are accepted. This research established a significant positive relationship between psychology and community engagement (β = 0.524, t = 12.780, p < 0.001), thus, H3 was supported. The results of the study demonstrate that education is a significant factor of community engagement (β = 0.476, t = 4.033, p< 0.001); hence, H4 was confirmed. The findings of the study indicate that education significantly influences positive youth development (β = 0.341, t = 7.577, p < 0.001), therefore, H5 was supported. Further, the result indicates that community engagement has a significant impact on positive youth development (β = 0.385, t = 3.235, p < 0.001), thus H6 was accepted.

**Table 5:** Hypotheses results.

Hypothesis	Estimate	S.E.	C.R.	P
H1: SW → CE	.563	.041	13.731	***
H2: SW → PYD	.631	.121	5.214	***
H3: PSY → CE	.524	.041	12.780	***
H4: ED → CE	.476	.118	4.033	***
H5: ED → PYD	.341	.045	7.577	***
H6: CE → PYD	.385	.119	3.235	***

*** p<0.001

**Fig 3:**
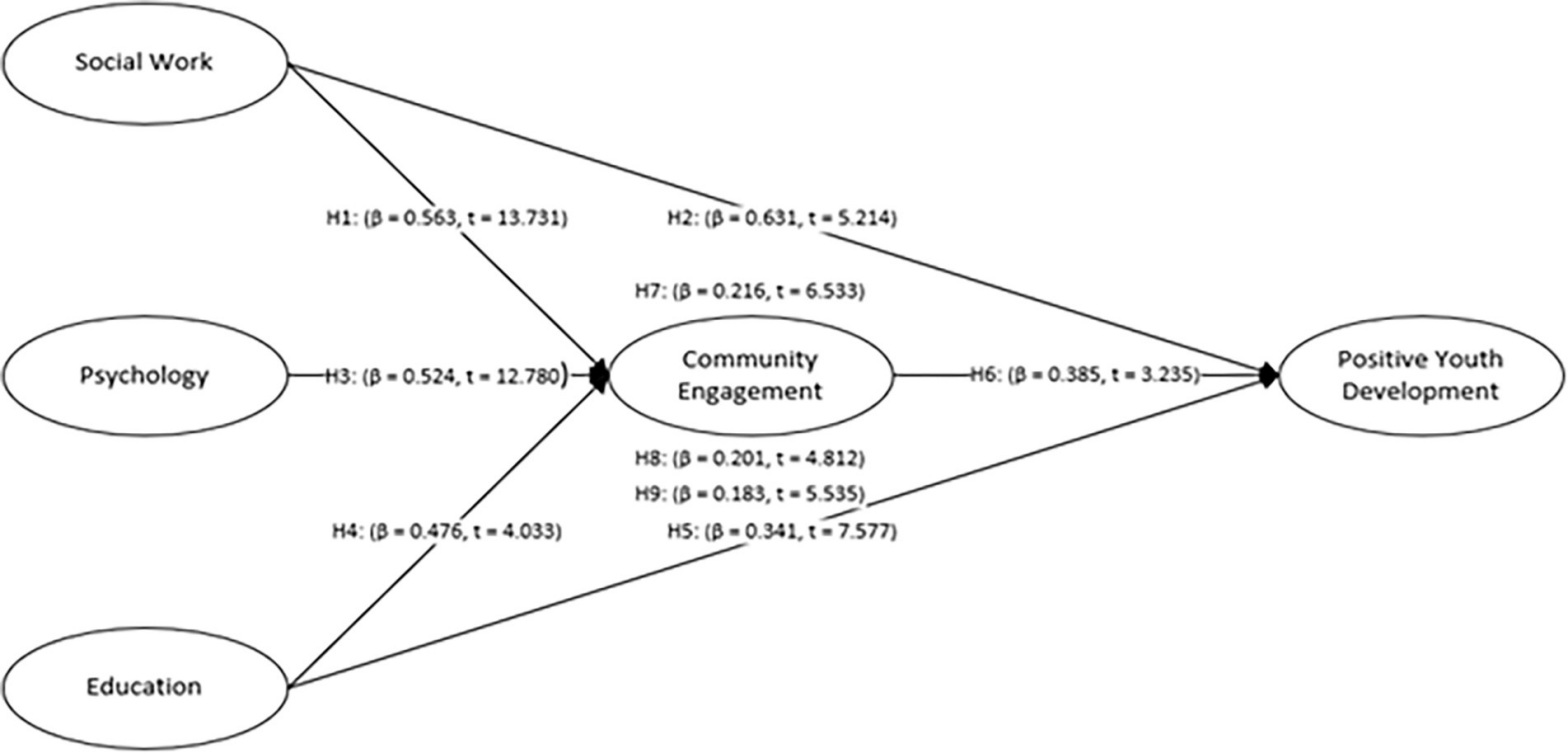
Path coefficient (direct and indirect effect).

The finding also shows that community engagement is an important mediating variable between social work and positive youth development (β = 0.216; p<0.05), psychology and positive youth development (β = 0.201; p<0.05), education and positive youth development (β = 0.183; p<0.05), thus H7, H8, and H9 were accepted. The path coefficient (direct and Indirect effect) is shown in Fig 3.

## 5. Discussion and conclusion

The main objective of the research was to explore the positive youth development in the Chinese rural area by integrating social work, psychology, and education with the community engagement as the mediating variable. The study findings show there is a positive relation between social work and the positive development for the youth of the Chinese rural communities, which proves H1. This implies that young people from rural areas opt either for social work intervention or core principles in order to be involved in the community directly [47]. Social interventions which may include community projects, support groups, mentoring, and workshops that cater for the rural young people might be envisioned. The results of the study revealed that social work plays a significant role in the development of youth in China, therefore supporting H2. For the betterment of the social and mental health of youth, social work initiatives can bring about such a huge difference by tackling social-economic problems, designing necessary programs, allocating resources effectively, and providing services. This denotes the incorporation of social work strategy in the youth development programs in order to facilitate positive youth results from the rural areas [65].

The study result shows that psychology is the main driver of community engagement; therefore, H3 is supported. Thus, self-efficacy, motivation, and social identity must be the core factors that can impact the youth of China to take part in societal affairs actively. The psychological interventions of facilitating a positive growth, behavioral cognitive change, and motivation are vital in encouraging community involvement among rural youth [66]. The findings indicate a strong relationship between education and community engagement among youth in rural China, thus supported H4. This demonstrates the way in which education can influence a person’s thinking patterns, action, and youth involvement in the society. When young people have access to high-quality education, they broaden their knowledge, they acquire new skills, and they develop the courage to be active members of the society [67]. Social responsibility among Chinese youth can be fostered through educational programs such as civic education, service- learning experiences, and community projects [68]. This therefore shows the great importance of education in developing civic participation through the process of cultivating responsible citizens.

The results of the study demonstrate that education significantly influences the positive youth development in rural communities of China, thus H5 was accepted. It highlights the fact that education is a transformative force that gives young people power and opportunity for a successful future. Through supply of formal education, vocational training, and life skilling development; educational programs address other vital areas of youth development that include academic performance, occupational readiness, and emotional health [69]. Investing in educational initiatives which meet the concrete needs of rural Chinese youth is the key for developing their holistic growth and for giving them access to the tools that will help them overcome the challenges that they will have to face. The findings of the study showed that community engagement found to be a strong factor that boosts positive youth development among rural young people in China, therefore H6 was supported. This signifies a positive way in which youth involvement in community activities have impact on their well-being and development. The process of being involved in the community, e.g., through volunteering, civic participation, and leadership, can provide social links, build skills, and strengthen resilience within youth [70]. Through encompassing active participation of youth in their communities, policymakers and practitioners in China may produce the environments that support youth development and help young people achieve their potential.

The results indicate that community engagement plays a crucial role in the connection between social work and positive youth development in the rural communities of China, thus accepting H7. Social work programs that encourage community participation not only give youngsters avenues to form social relationships, gain new skills, and enhance their community but also facilitate their growth into healthy, well-developed individuals. The findings of the study affirm the hypothesis 8 that community engagement acts as a mediator between psychology and positive youth development in the rural communities of China. Through emotional barriers identification and positive mindset development, psychological interventions promote the attainment of youth development outcomes. The testing of the hypothesis 9 in this study suggested that community engagement mediates the relationship between education and positive youth development in Chinese rural communities. Education provides community engaged programs that connect young people with their knowledge and skills in real-world situations, which enables personal development as well as social growth. The communicating role of community engagement highlights the relevance of educational programs that emphasize active learning, experiential education, and community-based approaches to strengthen holistic youth development in China.

## 6. Implications

### 6.1. Theoretical implications

This study has a theoretical contribution of the integrated approach that joins the perspectives of the social work, psychology, and education to promote the wellbeing of the youth. Through this research, the interconnection of the different disciplines is brought to the forefront, underlines the need for interdisciplinary collaboration when dealing with the complex and multi-layered socio-cultural factors that affect youth outcomes in the rural area. The findings imply the theoretical contribution of community engagement in the developmental process as one of the significant factors. Through the mediation role of community engagement, the study presents evidence that social work, psychology, education and the youth development act as major factors that impact the positive youth development. The research expands theoretical frameworks of youth development by highlighting the need for multi-dimensional approach which considers multiple components of mental health, such as social, emotional, and cognitive well-being. Through an analysis of the impact of social work, psychology and education on the favorable youth outcomes, the research broadens theoretical explanations of the processes underlying youth development in the rural context [71].

### 6.2. Practical implications

This research provides a practical base for policy makers, educators and practitioners who are involved in designing and implementing youth development programs in the remote regions. The study showcases the efficiency of social work, psychology, and education interventions in boosting community engagement and success of youth, which is vital in developing direct programs tailored for the specific needs of the youth in rural areas. The research contributes to the development of policies in rural areas targeting the well-being and development of youth [72]. Through the emphasis of the role of social work, psychology, and education in the promotion of community engagement and youth wellbeing, policymakers can therefore focus on giving priority to the programs and services that address the distinct issues of rural youth and at the same time facilitate the integration of these young people into wider socio-economic networks. The findings are a testament to the need of developing capacity-building programs that equip stakeholders working with rural youth with skills, knowledge, and resources they need. Through training and professional development, policymakers and organizations can give practitioners the needed tools to implement evidence-based interventions and to support positive youth outcomes in rural communities.

## 7. Limitations and future research

This study provides significant understanding about ways of helping youth to succeed in rural areas; however, the limitations exist. The use of convenience sampling and the reliance on subjective self-reported data may reduce the generalizability and may introduce response biases. The cross-sectional design also does not allow for causal inferences. In addition, future research can be based on a more rigorous sampling such as stratified or random sampling, and use longitudinal approach to establish causality. Qualitative studies could provide insights into youth experiences that would be otherwise unavailable. Comparative study from different cultural contexts might clarify the existence of universal or the special nature of the findings. Through the recognition and investigation of these constraints and other similar studies it would contribute to our understanding of the complementary and conflictive relationships that social work, psychology, education, community engagement and positive youth development possess in rural areas.

## References

[1] Su S., Li X., Lin D., Zhu M. (2017). Future orientation, social support, and psychological adjustment among left-behind children in rural China: A longitudinal study. Frontiers in Psychology, 8, 265942. doi: 10.3389/fpsyg.2017.01309 28824493 PMC5539116

[2] Lerner R. M., Lerner J. V., Murry V. M., Smith E. P., Bowers E. P., Geldhof G. J., et al. (2021). Positive youth development in 2020: Theory, research, programs, and the promotion of social justice. Journal of Research on Adolescence, 31(4), 1114–1134. doi: 10.1111/jora.12609 34820946

[3] Wang G. (2021). ‘They are bad seeds’: stereotyping habitus in Chinese VET colleges. British Journal of sociology of Education, 42(7), 1008–1021.

[4] Shi W., Shen Z., Wang S., Hall B. J. (2020). Barriers to professional mental health help-seeking among Chinese adults: a systematic review. Frontiers in psychiatry, 11, 442. doi: 10.3389/fpsyt.2020.00442 32508688 PMC7251144

[5] Leng X., Zhong M., Xu J., Xie S. (2020). Falling into the second-generation decline? Evidence from the intergenerational differences in social identity of rural–urban migrants in China. SAGE Open, 10(3), 2158244020939539.

[6] Ungar M. (2020). Working with children and youth with complex needs: 20 skills to build resilience. Routledge.

[7] Galagali P. M., Brooks M. J. (2020). Psychological care in low-resource settings for adolescents. Clinical child psychology and psychiatry, 25(3), 698–711. doi: 10.1177/1359104520929741 32567351 PMC9137117

[8] Ennin F., Pushpanadham K. (2023). African Universities and Industry Partnership for Quality Higher Education: Global Framework for Graduate Attributes. International Journal of Scientific Research and Innovative Studies, 2(4), 09–18.

[9] Harsanto B. T., Wahyuningrat W. (2024). Investigating the keys to the failure of inter-village government collaboration in developing rural economic potentials in Indonesia. Regional Science Policy & Practice, 16(5), 100023.

[10] Dang L., Seemann A. K., Lindenmeier J., Saliterer I. (2022). Explaining civic engagement: The role of neighborhood ties, place attachment, and civic responsibility. Journal of community psychology, 50(3), 1736–1755. doi: 10.1002/jcop.22751 34807467 PMC9298745

[11] Eklund M., Khalilpour K., Voinov A., Hossain M. (2023). Understanding the community in community microgrids: A conceptual framework for better decision-making. Energy Research & Social Science, 104, 103260.

[12] Deb S., Deb S. (2023). Positive Youth Development Through Holistic Approach. In Handbook of Youth Development: Policies and Perspectives from India and Beyond (pp. 3–34). Singapore: Springer Nature Singapore.

[13] Wu H., Greig M., Bryan C. (2022). Promoting environmental justice and sustainability in social work practice in rural community: A systematic review. Social Sciences, 11(8), 336.

[14] Morse D. F., Sandhu S., Mulligan K., Tierney S., Polley M., Giurca B. C., et al. (2022). Global developments in social prescribing. BMJ Global Health, 7(5), e008524. doi: 10.1136/bmjgh-2022-008524 35577392 PMC9115027

[15] Nyahunda L., Chibvura S., Tirivangasi H. M. (2021). Social work practice: accounting for double injustices experienced by women under the confluence of Covid-19 pandemic and climate change impacts in Nyanga, Zimbabwe. Journal of Human Rights and Social Work, 6(3), 213–224. doi: 10.1007/s41134-021-00170-4 34056061 PMC8150630

[16] Rošker J. S. (2020). Modern new Confucianism and the challenges of Chinese modernity: intercultural dialogues in Chinese philosophy. Culture and Dialogue, 8(2), 196–219.

[17] Shu Q., Wang Y. (2021). Collaborative leadership, collective action, and community governance against public health crises under uncertainty: a case study of the Quanjingwan community in China. International Journal of Environmental Research and Public Health, 18(2), 598. doi: 10.3390/ijerph18020598 33445697 PMC7828130

[18] Senapati R. E., Jena S., Parida J., Panda A., Patra P. K., Pati S., et al. (2024). The patterns, trends and major risk factors of suicide among Indian adolescents–a scoping review. BMC Psychiatry, 24(1), 35. doi: 10.1186/s12888-023-05447-8 38195413 PMC10775453

[19] Williams T., Lakhani A., Spelten E. (2022). Interventions to reduce loneliness and social isolation in rural settings: A mixed-methods review. Journal of rural studies, 90, 76–92.

[20] Javed A., Lee C., Zakaria H., Buenaventura R. D., Cetkovich-Bakmas M., Duailibi K., et al. (2021). Reducing the stigma of mental health disorders with a focus on low-and middle-income countries. Asian journal of psychiatry, 58, 102601. doi: 10.1016/j.ajp.2021.102601 33611083

[21] Ahmad F., Chowdhury R., Siedler B., Odek W. (2022). Building community resilience during COVID‐19: Learning from rural Bangladesh. Journal of Contingencies and Crisis Management, 30(3), 327–338. 10.1111/1468-5973.12405 PMC9111107 40478069

[22] Schafft K. A. (2016). Rural education as rural development: Understanding the rural school–community well-being linkage in a 21st-century policy context. Peabody Journal of Education, 91(2), 137–154.

[23] Jackson K. T., Burgess S., Toms F., Cuthbertson E. L. (2018). Community engagement: Using feedback loops to empower residents and influence systemic change in culturally diverse communities. Global Journal of Community Psychology Practice, 9(2), 1–21.

[24] Anselma M., Chinapaw M., Altenburg T. (2020). Not only adults can make good decisions, we as children can do that as well” evaluating the process of the youth-led participatory action research ‘kids in action. International journal of environmental research and public health, 17(2), 625. doi: 10.3390/ijerph17020625 31963706 PMC7014142

[25] McFadden P., Ross J., Moriarty J., Mallett J., Schroder H., Ravalier J., et al. (2021). The role of coping in the wellbeing and work-related quality of life of UK health and social care workers during COVID-19. International journal of environmental research and public health, 18(2), 815. doi: 10.3390/ijerph18020815 33477880 PMC7832874

[26] Aguilar-Gaxiola S., Ahmed S. M., Anise A., Azzahir A., Baker K. E., Cupito A., et al. (2022). Assessing meaningful community engagement: a conceptual model to advance health equity through transformed systems for health: organizing committee for assessing meaningful community engagement in health & health care programs & policies. NAM Perspectives, 2022, doi: 10.31478/202202c 35891775 PMC9303007

[27] Woolcott G., Loosemore M., Keast R., Metzer A., Alkilani S. (2024). Transitioning young people into employment in the Australian construction industry: the trust-building role of project-based intermediaries. Engineering, Construction and Architectural Management.

[28] Wu H. (2021). Integration of the disaster component into social work curriculum: Teaching undergraduate social work research methods course during COVID-19. The British Journal of Social Work, 51(5), 1799–1819.

[29] Dimitrova R., Wiium N. (2021). Handbook of positive youth development: Advancing the next generation of research, policy and practice in global contexts. Springer.

[30] Moors M., Osis J. (2024). Changes in the approach of social work with young people in out-of-home care in Riga. SHS Web of Conferences.

[31] Sulimani‐Aidan Y., Melkman E. (2022). School belonging and hope among at‐risk youth: The contribution of academic support provided by youths’ social support networks. Child & Family Social Work, 27(4), 700–710.

[32] Germain C., Knight C. (2020). The life model of social work practice: Advances in theory and practice. Columbia University Press.

[33] Damon W., Lerner R. M., Eisenberg N. (2006). Handbook of child psychology, social, emotional, and personality development. John Wiley & Sons.

[34] Eklund K., DeMarchena S. L., Rossen E., Izumi J. T., Vaillancourt K., Rader Kelly S. (2020). Examining the role of school psychologists as providers of mental and behavioral health services. Psychology in the Schools, 57(4), 489–501.

[35] Killaspy H., Harvey C., Brasier C., Brophy L., Ennals P., Fletcher J., et al. (2022). Community‐based social interventions for people with severe mental illness: a systematic review and narrative synthesis of recent evidence. World Psychiatry, 21(1), 96–123. doi: 10.1002/wps.20940 35015358 PMC8751572

[36] Zikargae M. H., Woldearegay A. G., Skjerdal T. (2022). Assessing the roles of stakeholders in community projects on environmental security and livelihood of impoverished rural society: A nongovernmental organization implementation strategy in focus. Heliyon, 8(10), e10987. doi: 10.1016/j.heliyon.2022.e10987 36276717 PMC9582698

[37] Madsen W., Ambrens M., Ohl M. (2019). Enhancing resilience in community-dwelling older adults: a rapid review of the evidence and implications for public health practitioners. Frontiers in public health, 7, 422850. doi: 10.3389/fpubh.2019.00014 30792974 PMC6374312

[38] Ismail I., Ali H., Us K. A. (2022). Factors Affecting Critical and Holistic Thinking in Islamic Education in Indonesia: Self-Concept, System, Tradition, Culture.(Literature Review of Islamic Education Management). Dinasti International Journal of Management Science, 3(3), 407–437.

[39] Papp T. A. (2020). Teacher Practices and Professional Development that Promote Improved Educational Outcomes for Indigenous Students in Saskatchewan and New Zealand (Doctoral dissertation, University of Saskatchewan).

[40] Akar B. (2016). Dialogic pedagogies in educational settings for active citizenship, social cohesion and peacebuilding in Lebanon. Education, citizenship and social justice, 11(1), 44–62.

[41] Evans M. P. (2018). Educating preservice teachers for family, school, and community engagement. Family, School, Community Engagement and Partnerships, 9–19.

[42] Shirley D. (2020). Beyond well-being: The quest for wholeness and purpose in education. ECNU Review of Education, 3(3), 542–555.

[43] Holmlund H., Nybom M. (2023). Education and social mobility (No. 2023: 18). Working Paper.

[44] Allaste A.-A., Beilmann M., Pirk R. (2022). Non-formal and informal learning as citizenship education: The views of young people and youth policymakers. Journal of Applied Youth Studies, 5(1), 19–35.

[45] Frimpomaa-Afrane A. (2023). Towards a better Ghana?: understanding barriers and facilitators to youth development from the perspectives of students in higher education.

[46] Jelicic H., Bobek D. L., Phelps E., Lerner R. M., Lerner J. V. (2021). Using positive youth development to predict contribution and risk behaviors in early adolescence: Findings from the first two waves of the 4-H Study of Positive Youth Development. In Individuals as Producers of Their Own Development (pp. 204–225). Routledge.

[47] Trivelli C., Morel J. (2021). Rural youth inclusion, empowerment, and participation. The Journal of Development Studies, 57(4), 635–649.

[48] Barnason S., Li C. J., Hall D. M., Wilhelm Stanis S. A., Schulz J. H. (2022). Environmental action programs using positive youth development may increase civic engagement. Sustainability, 14(11), 6781.

[49] Kjellander T., Sjöblom L. (2023). Child and youth participation during crisis: Recommendations for decision makers in the Nordic region. In: Nordens välfärdscenter/Nordic Welfare Centre.

[50] Sharkey C. N. (2023). Digital Storytelling and Fostering Collective Efficacy: Arts-Based Youth Participatory Action Research and Constructing Community (Doctoral dissertation, University of Georgia).

[51] Smith E. P., Yunes M. A. M., Fradkin C. (2021). From prevention and intervention research to promotion of positive youth development: Implications for global research, policy and practice with ethnically diverse youth. Handbook of positive youth development: Advancing research, policy, and practice in global contexts, 549–566.

[52] García-Romero D., Martínez-Lozano V. (2022). Social participation and theoretical content: Appropriation of curricular concepts in service-learning. Journal of Higher Education Outreach and Engagement, 26(1), 71–87.

[53] Zhang Z., Yu J., Tian J. (2023). Community Participation, Social Capital Cultivation and Sustainable Community Renewal: A Case Study from Xi’an’s Southern Suburbs, China. Journal of the Knowledge Economy, 1–34.

[54] Mahoney J. L., Weissberg R. P., Greenberg M. T., Dusenbury L., Jagers R. J., Niemi K., et al. (2021). Systemic social and emotional learning: Promoting educational success for all preschool to high school students. American Psychologist, 76(7), 1128. doi: 10.1037/amp0000701 33030926

[55] Hurd A. B. (2020). Focus on Youth: Awakening Youth Voice & Engagement in Community Heritage through the Implementation of a Youth Participatory Empowerment Model. University of Missouri-Saint Louis.

[56] Thakkar J. J. (2020). Structural equation modelling. Application for Research and Practice. Springer.

[57] Hair J., Hult G.T.M., Ringle C., Sarstedt M., (2016). A Primer on Partial Least Squares Structural Equation Modeling (PLS-SEM). Sage, London, UK.

[58] Bentler P. M. (1990). Comparative fit indexes in structural models. Psychological bulletin, 107(2), 238. doi: 10.1037/0033-2909.107.2.238 2320703

[59] Hu L.-t., Bentler P. M. (1998). Fit indices in covariance structure modeling: Sensitivity to underparameterized model misspecification. Psychological methods, 3(4), 424.

[60] Nunnally J., Bernstein I. (1994). The assessment of reliability. Psychometric Theory, 3 (1), 248–292.

[61] Hair J., Black W.C., Babin B.J., Anderson R.E., Tatham R. (2010). Multivariate Data Analysis, 7th ed. Prentice Hall, Upper Saddle River, NJ.

[62] Fornell C., Larcker D. F. (1981). Structural equation models with unobservable variables and measurement error: Algebra and statistics. In: Sage publications Sage CA: Los Angeles, CA.

[63] Henseler J., Ringle C. M., Sarstedt M. (2015). A new criterion for assessing discriminant validity in variance-based structural equation modeling. Journal of the academy of marketing science, 43, 115–135.

[64] Gold A. H., Malhotra A., Segars A. H. (2001). Knowledge management: An organizational capabilities perspective. Journal of management information systems, 18(1), 185–214.

[65] Goodman M. L., Seidel S. E., Springer A., Elliott A., Markham C., Serag H., et al. (2023). Enabling structural resilience of street-involved children and youth in Kenya: reintegration outcomes and the flourishing community model. Frontiers in Psychology, 14, 1175593. doi: 10.3389/fpsyg.2023.1175593 37680240 PMC10482225

[66] Njau T., Ngakongwa F., Sunguya B., Kaaya S., Fekadu A. (2022). Development of a Psychological Intervention to Improve Depressive Symptoms and Enhance Adherence to Antiretroviral Therapy among Adolescents and Young People Living with HIV in Dar es Salaam Tanzania. Healthcare (Basel, Switzerland), 10(12), 2491. doi: 10.3390/healthcare10122491 36554015 PMC9778412

[67] Szymkowiak A., Melović B., Dabić M., Jeganathan K., Kundi G. S. (2021). Information technology and Gen Z: The role of teachers, the internet, and technology in the education of young people. Technology in Society, 65, 101565.

[68] Sze-Yeung Lai C., & Chi-leung Hui P. (2021). Service-learning: Impacts of learning motivation and learning experience on extended social/civic engagement. Higher Education Research & Development, 40(2), 400–415.

[69] Ochieng V. O., Ngware M. (2022). Whole youth development and employment: Exploring the nexus using qualitative data from a Kenyan study of Technical and Vocational Education and Training institutions. Journal of Adult and Continuing Education, 28(2), 558–594.

[70] Chan W. Y., Ou S.-R., Reynolds A. J. (2014). Adolescent civic engagement and adult outcomes: An examination among urban racial minorities. Journal of youth and adolescence, 43, 1829–1843. doi: 10.1007/s10964-014-0136-5 24878896 PMC4192036

[71] Paricio D., Herrera M., Rodrigo M. F., Viguer P. (2020). Association between group identification at school and positive youth development: Moderating role of rural and urban contexts. Frontiers in Psychology, 11, 555017. doi: 10.3389/fpsyg.2020.01971 32849154 PMC7427468

[72] Branquinho C., Tomé G., Grothausen T., Gaspar de Matos M. (2020). Community‐based Youth Participatory Action Research studies with a focus on youth health and well‐being: A systematic review. Journal of community psychology, 48(5), 1301–1315. doi: 10.1002/jcop.22320 31985839

